# Machine Learning Diagnostic Model for Hepatocellular Carcinoma Based on Liquid–Liquid Phase Separation and Ferroptosis-Related Genes

**DOI:** 10.5152/tjg.2024.24101

**Published:** 2024-10-07

**Authors:** Wenchao Chen, Ting Zhu, Xiaofan Pu, Linlin Zhao, Senhao Zhou, Xin Zhong, Suihan Wang, Tianyu Lin

**Affiliations:** 1 Department of General Surgery, Zhejiang University School of Medicine, Sir Run Run Shaw Hospital, Hangzhou, China; 2 Department of Thoracic Surgery, Shaoxing People’s Hospital, Shaoxing, China; 3 Department of Cardiology, Zhejiang University School of Medicine, Sir Run Run Shaw Hospital, Hangzhou, China; 4 Department of Otolaryngology Head and Neck Surgery, Zhejiang University School of Medicine, Sir Run Run Shaw Hospital, Hangzhou, China

**Keywords:** Hepatocellular carcinoma, liquid–liquid phase separation, ferroptosis, diagnostic model, survival

## Abstract

**Background/Aims:**

Hepatocellular carcinoma (HCC) represents a primary liver malignancy with a multifaceted molecular landscape. The interplay between liquid–liquid phase separation (LLPS) and ferroptosis—a regulated form of cell death—has garnered interest in tumorigenesis. However, the precise role of LLPS and ferroptosis-related genes in HCC progression and prognosis remains obscure. Unraveling this connection could pave the way for innovative diagnosis and therapeutic strategies.

**Materials and Methods:**

The differentially expressed genes (DEGs) were identified based on 3 GEO datasets, followed by overlapping with LLPS-related and ferroptosis-related genes. Based on central hub genes, a diagnostic model was developed through LASSO regression and validated using KM survival analysis and real-time quantitative polymerase chain reaction (RT-qPCR). Then the effects of NRAS on the development of HCC and ferroptosis were also detected.

**Results:**

We identified 24 DEGs overlapping among HCC-specific, LLPS, and ferroptosis-related genes. A diagnostic model, centered on 5 hub genes, was developed and validated. Lower expression of these genes corresponded with enhanced patient survival rates, and they were distinctly overexpressed in HCC cells. NRAS downregulation significantly inhibited HepG2 cell proliferation and migration (*P* < .01). Fe^2+^ content and ROS levels were both significantly increased in the si-NRAS group when compared to those in the si-NC group (*P* < .01), while opposite results were observed for the protein level of GPX4 and GSH content.

**Conclusion:**

The diagnostic model with 5 hub genes (EZH2, HSPB1, NRAS, RPL8, and SUV39H1) emerges as a potential innovative tool for the diagnosis of HCC. NRAS promotes the carcinogenesis of HCC cells and inhibits ferroptosis.

**Main Points**

- Liquid–liquid phase separation and ferroptosis genes shape the molecular landscape of HCC.
- Five hub genes (EZH2, HSPB1, NRAS, RPL8, and SUV39H1) form a novel HCC diagnostic model.
- Lower expression of signature genes corresponded with enhanced patient survival rates.
- Signature genes were distinctly overexpressed in HCC cells.

## Introduction

**Supplementary Figure 1.**
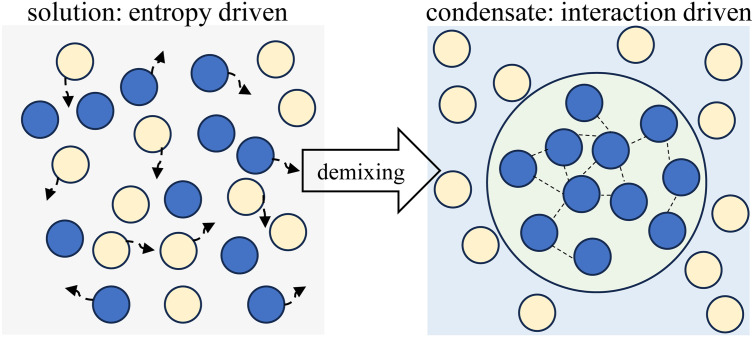
liquid-liquid phase separation (LLPS). Whereas entropy typically drives molecules to become dispersed in solution, mutual interactions among a subset of molecules can shift the free-energy landscape to favor demixing and drive the formation of a separate condensed phase.

Liver cancer presents a significant global health concern, with projections indicating that over 1 million people will be affected annually by 2025. Hepatocellular carcinoma (HCC), constituting 90% of primary liver cancers, ranks as the fourth primary cause of cancer-related mortalities globally.^1^ The development and progression of HCC are influenced by a complex interplay of factors. These include genetic predispositions, both viral and non-viral risk factors like fatty liver disease, immune responses, and the tumor microenvironment.^1^ The concept of liquid–liquid phase separation (LLPS) involving proteins and nucleic acids has recently come to the forefront in cellular biology research. Liquid–liquid phase separation describes the spontaneous de-mixing of a homogeneous solution into 2 or more distinct phases. Phase separation occurs when interactions among groups of like molecules overcome the tendency to remain disordered in solution (i.e., entropy) (Supplementary Figure 1).^2,3^ This mechanism facilitates the formation of membrane-less, liquid-like condensates within living cells, encapsulating specific biomolecules.^4^ Liquid–liquid phase separation underpins the creation of signaling condensates, thereby modulating immune signaling pathways.^5^ Also, LLPS plays a pivotal role in various cellular processes, encompassing transcriptional regulation, ensuring genome stability, and facilitating signal transduction. Its involvement might also have implications in the onset of tumorigenesis and the progression of tumors.^4^

Liquid–liquid phase separation has been implicated in instigating and driving cancer progression through several mechanisms, including transcription, cell signaling, and DNA repair.^6,7^ For instance, Chen et al^8^ demonstrated that circVAMP3 inhibits the proliferation and metastasis of HCC cells by promoting the phase separation of CAPRIN1. Liu et al^9^ revealed that LLPS mediated by circRNA-YBX1 facilitates cytoskeletal remodeling, consequently attenuating the metastatic potential of liver cancer. Zhang et al^10^ demonstrated that a PKA fusion oncoprotein tied to atypical liver cancer disrupts RIα LLPS, resulting in aberrant cAMP signaling and heightened cell proliferation and transformation. Li et al^11^ uncovered that the Smad2/3/4 complex could undergo LLPS and induce apoptosis through tyrosine aminotransferase in HCC. Liu et al^12^ revealed that LLPS of glycogen encapsulates YAP into glycogen droplets, inhibits the activation of the Hippo pathway and drives the occurrence of HCC. Meng et al^13^ illustrated that the Twist1-YY1-p300 complex promotes miR-9 expression through LLPS, stimulating HCC cell invasion and metastasis. Collectively, these investigations elucidate the potential significance of LLPS in HCC pathogenesis, offering profound insights into its molecular underpinnings and informing potential diagnostic and therapeutic strategies.

Within the distinct environment of the LLPS droplet, pathological protein aggregation is notably enhanced, contributing to ferroptosis.^14^ Ferroptosis, a recently recognized cell death mode marked by iron accumulation and lipid peroxidation, has surfaced as a promising target for anticancer therapy.^15,16^ Studies have indicated that elevated ferroptosis levels enhance the radiosensitivity of HCC.^17,18^ Gao et al^19^ uncovered that YAP/TAZ and ATF4 mediate resistance to Sorafenib in HCC by inhibiting ferroptosis. A study found that in the TCGA cohort, 81.7% of ferroptosis-related genes showed differential expression between HCC and adjacent normal tissues.^20^ In addition, it is reported that ferroptosis is involved in sorafenib resistance in HCC.^21,22^ In HCC patients progressing under first-line sorafenib therapy, treatment with metronomic capecitabine, which is one of the agents able to induce ferroptosis,^23^ demonstrated a very good efficacy and safety profile.^24^ These findings suggest that targeting ferroptosis might offer a therapeutic avenue for HCC. In addition, it has been reported that LLPS, responsible for the aberrant accumulation of α-syn and tau, in conjunction with iron metabolism dysfunction, is the central driver of ferroptosis.^14^ Through phase separation or abnormal phase separation, tumor-related biological macromolecules, such as mRNA, long noncoding RNAs (lncRNAs), and tumor-related proteins, can affect transcriptional translation and DNA damage repair, regulate the autophagy and ferroptosis functions of cells, and thus regulate the development of various tumors.^25^ Li et al^26^ revealed that EphA2 is a phase separation protein associated with ferroptosis and immune cell infiltration in colorectal cancer. This discovery suggests a potential link between LLPS and ferroptosis in HCC. However, few studies have reported LLPS and ferroptosis in HCC. Further research is essential to elucidate the interplay and molecular mechanisms connecting LLPS and ferroptosis, paving the way for innovative therapeutic approaches for HCC.

In this study, we identified LLPS and ferroptosis-relevant differentially expressed genes (DEGs) by analyzing data of HCC patients from public databases. According to the identified hub genes, we created a diagnostic model to predict patients with HCC. Our research will guide clinicians in tailoring treatments for HCC patients, potentially enhancing their prognosis.

## Materials and Methods

### Data Acquisition and pre-Processing

We retrieved gene expression datasets GSE45267, GSE65372, GSE84402, and GSE76427 associated with HCC from the Gene Expression Omnibus (GEO) database (https://www.ncbi.nlm.nih.gov/geo/). These datasets encompassed samples from 216 HCC patients and 120 controls. Details of each dataset, including sample descriptions, are provided in Table 1. In this study, the GSE76427 dataset was used as an external validation set. Probes were annotated utilizing the SOFT formatted family file(s) provided on the GEO platform, converting each probe to its corresponding gene symbol. It is essential to acknowledge that batch effects, which can introduce significant variability in scientific studies, may emanate from differences in experimental conditions, methodologies, or materials. To mitigate these effects, we utilized the “sva” package in R (version 4.2.2, University of Auckland, Auckland, New Zealand) to integrate the 3 datasets, effectively removing batch effects. Furthermore, we conducted principal component analysis (PCA) to reduce dimensionality and evaluate the efficacy of our batch effect corrections.

### Differential Gene Expression Analysis in Hepatocellular Carcinoma

Employing R (version 4.2.2), we utilized the “limma” package^27^ to pinpoint DEGs between HCC and control samples, adopting the criteria of *P*-value < .05 and an absolute log2 fold change (|log2FC|) exceeding 0.5. For visualization, DEGs were represented through volcano plots, hierarchical clustering heatmaps, and PCA plots, all curated using the “ggplot2” package in R.

### Identification of Liquid–Liquid Phase Separation and Ferroptosis Genes

We procured 3773 distinct LLPS genes from the DrLLPS (http://llps.biocuckoo.cn/) and PhaSepDB (http://db.phasep.pro/) databases. FerrDb (http://www.zhounan.org/ferrdb/current/) serves as a curated repository, cataloging ferroptosis regulatory elements in both humans and mice. From FerrDb, we amassed a compilation of 396 genes, encompassing drivers, suppressors, markers, and regulators awaiting classification. To discern the overlap between the LLPS and ferroptosis genes, we employed the “VennDiagram” package in R.

### Protein–Protein Interaction Network Construction

The overlapping genes were introduced into the STRING database (https://string-db.org/) for PPI network generation. Only interactions boasting a combined score exceeding 0.4 were retained. This network was subsequently visualized using Cytoscape, with the 15 most connected genes identified, based on their nodal degree, to serve as central nodes in the regulatory framework.

### Diagnostic Model Construction and Validation

LASSO regression, introduced by Robert Tibshirani in 1996, is a technique that contracts regression coefficients and can zero out irrelevant predictors.^28^ To guard against overfitting, we allocated 60% of our dataset samples to a training set. Using the foremost 15 genes derived from the PPI network, we devised a diagnostic model via LASSO regression. Herein, the LASSO regression analysis was performed with 10-fold cross-validation to select feature genes via the binomial method in the glmnet package, and the feature genes were identified with the parameter of lambda.1se, nlambda = 50, alpha = 1. We evaluated the predictive accuracy of the model on the residual 40% validation dataset using the “pROC” package in R.

### Prognostic Validation of Signatures

To explore the clinical significance of the biomarker genes, we assessed their association with survival using GEPIA (http://gepia.cancer-pku.cn/detail.php). Kaplan-Meier survival analyses were performed to analyze the association between gene expression and the survival of HCC patients. The significance of differences in survival was determined through a log-rank test.

### Cell Culture and Transfection

The human normal hepatic epithelial cell line THLE-3 (CTCC-001-0067, Zhejiang Meisen Cell Technology Co., Ltd., China) and HCC cell lines HepG2 (CTCC-001-0014), Huh7 (CTCC-003-0019), and Hep3B (YS487C, Shanghai Yaji Biotechnology Co., Ltd., China) were utilized for the study. THLE-3 cells were cultured in BEGM medium (Lonza, Switzerland) enriched with 10% fetal bovine serum (FBS; Gibco, USA), 100 U/mL penicillin, 100 μg/mL streptomycin (HyClone, USA), and 5 ng/mL EGF (MCE, China). HepG2 and Huh7 cells were maintained in DMEM (Gibco) supplemented with 10% FBS, 100 U/mL penicillin, and 100 μg/mL streptomycin. In contrast, Hep3B cells thrived in MEM (Gibco) containing the same additives. All cells were incubated at 37°C in a 5% CO_2_ humidified atmosphere, with media replacements every 2-3 days. Upon achieving 80%-90% confluency, routine passaging was conducted. To observe the effect of NRAS, the lentiviral vector of NRAS (si-NRAS) was constructed and transfected into HepG2 cells to interfere with the NRAS expression. The following siRNA sequences were designed from the Designer of Small Interfering RNA website: si-NRAS: 5’-CACTTTGTAGATGAATATGATCC-3’, 5’-GGATCATATTCATCTACAAAGTG-3’.

### Cell Counting Kit 8 Assay

Cell proliferation was assessed using the CCK-8 kit (C0037, Beyotime, China). At 48 hours after transfection, the absorbance at 450 nm was detected using a microplate reader (DR-3518G, Wuxi Hiwell Diatek, China). Each experiment was repeated 3 times.

### Transwell

The treated HepG2 cells were diluted to 10 × 10^5^/mL with high sugar DMEM basic medium, and 600 μL complete DMEM medium containing 20% FBS was added to the lower chamber, and 200 μL cell suspension was added to the lower chamber. The 24-well plate with Transwell chamber was incubated in a 37°C incubator for 24 hours. After 24 hours, the liquid was cleaned in a hole containing 600 μL PBS 3 times. After crystal violet staining for 20 minutes, an inverted microscope (DMi3000 B, Leica Microsystems Inc., Germany) was used for capturing images.

### Western Blot Assay

The treated HepG2 cells were lysed for 30 minutes, separated by SDS-PAGE, and then transferred to a polyvinylidene fluoride (PVDF) membrane (FFP24, Beyotime, China). After sealing with 3% BSA, primary antibodies against GPX4 (1:1000; ab125066; Abcam, USA) and GAPDH (1:1000; ab181602; Abcam, USA) were added overnight at 4°C, and the corresponding peroxidase-labeled secondary antibody was added. The levels of GPX4 were evaluated according to the Electrochemical luminescence (ECL) reagents (34579, Pierce, USA).

### Real-Time Quantitative Polymerase Chain Reaction

Total RNA from cells was isolated using the TRIzol® Reagent (Invitrogen, USA). From this, 1 μg of total RNA was reverse transcribed to cDNA in a 20 μL reaction using the FastKing One-Step Genomic DNA Removal and First-Strand cDNA Synthesis Premix (TIANGEN, China). Real-time quantitative polymerase chain reaction assays were conducted with the SYBR Green PCR Master Mix (Lifeint, China) on the CFX96 Touch Real-Time PCR Detection System (Bio-Rad, USA). Relative mRNA expression of target genes was calibrated to GAPDH and quantified using the 2^−∆∆CT^ method. Each RT-qPCR assay was executed in triplicate, and the entire procedure was independently replicated 3 times. Primer sequences are provided in Table 2.

### Reactive Oxygen Species and Glutathione and Iron Assay

Intracellular ferrous iron (Fe^2+^) content was measured by Fe^2+^ detection reagent kit (MAK025 Meack, Germany). The GSH level was detected using a GSH detection reagent kit (S0053, Beyotime, China). The ROS level was measured by the Reactive Oxygen Species Assay Kit (S0033S, Beyotime, China), and the results were detected using a CytoFLEX S flow cytometer (Beckman Coulter, USA).

### Statistical Analysis

All data are presented as mean ± SD. Differences between groups were assessed using 1-way analysis of variance (ANOVA). Statistical analyses were performed using GraphPad Prism 7.0 software (San Diego, CA, USA). A *P*-value of <.05 was considered statistically significant.

## Results

### Identification of Differentially Expressed Genes in Hepatocellular Carcinoma

**Figure 1.**
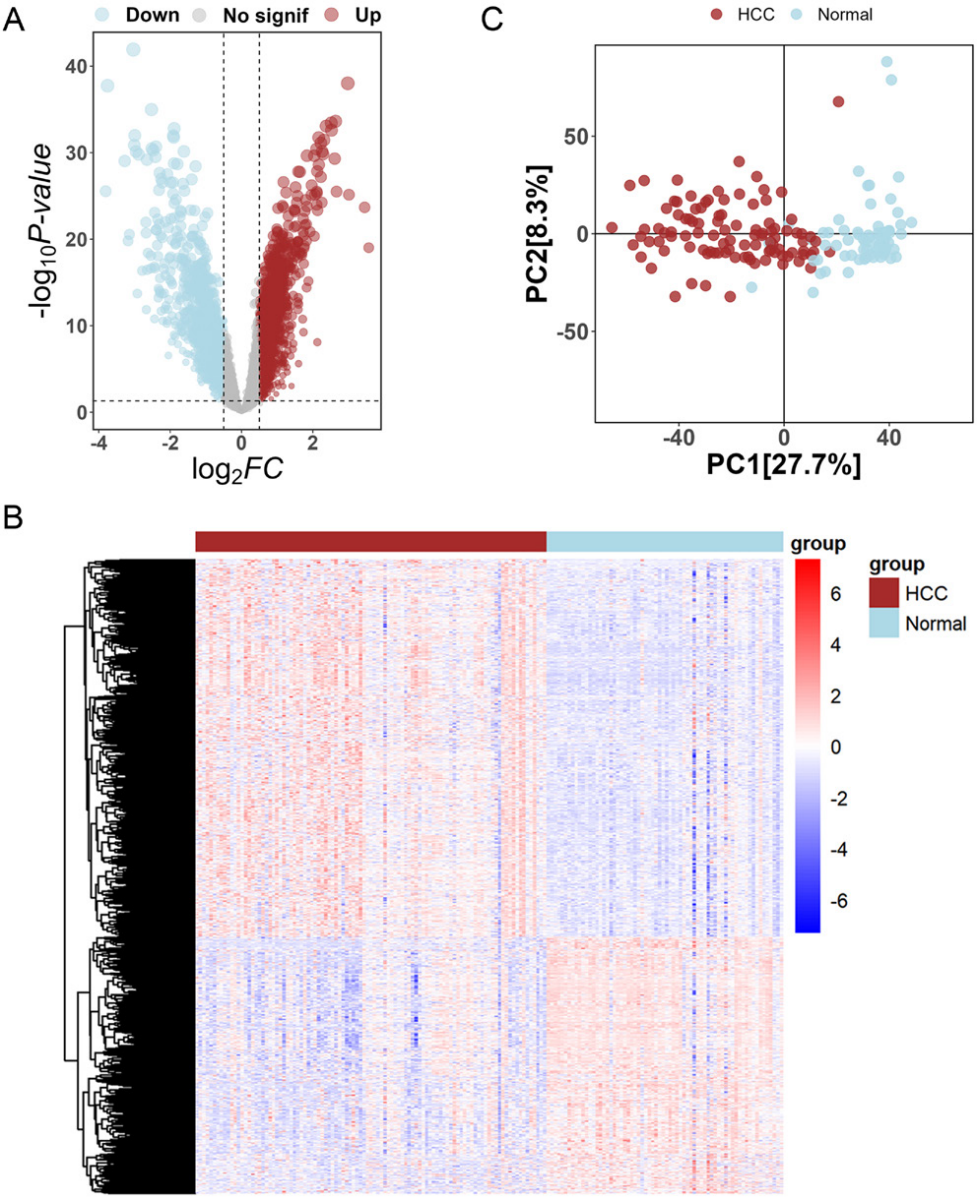
Differential expression analysis in HCC vs. normal samples. (a) Volcano plots highlighting the 3286 DEGs based on a threshold of |log2FC| > .5 and *P*-value < .05. (b) Heatmap depicting expression patterns of identified DEGs. (c) PCA analysis illustrating clear clustering differentiation of DEGs between HCC and normal samples.

We employed the limma package to identify DEGs in each of the 3 HCC–GEO datasets (GSE45267, GSE65372, and GSE84402). Before data integration, there was a marked distinction in the distributions of the 3 datasets following PCA dimensionality reduction, pointing to a pronounced batch effect (Supplementary Figure 1a). However, after rectifying the batch effects, the datasets exhibited a more homogeneous distribution in the PCA-reduced space, indicating successful mitigation of batch effect influences on the gene expression profiles (Supplementary Figure 1b). Applying a threshold of |log2FC| > 0.5 and a *P*-value < .05, we discerned 3286 DEGs between HCC and normal samples across the 3 datasets, with 1953 up-regulated and 1333 down-regulated. Their corresponding volcano plots and heatmap are illustrated in Figure 1a and b. Following the PCA analysis of DEGs, we identified a pronounced differentiation in clustering patterns between HCC and normal samples (Figure 1c).

### Identification of Liquid–Liquid Phase Separation and Ferroptosis-Related Differentially Expressed Genes in Hepatocellular Carcinoma

**Figure 2.**
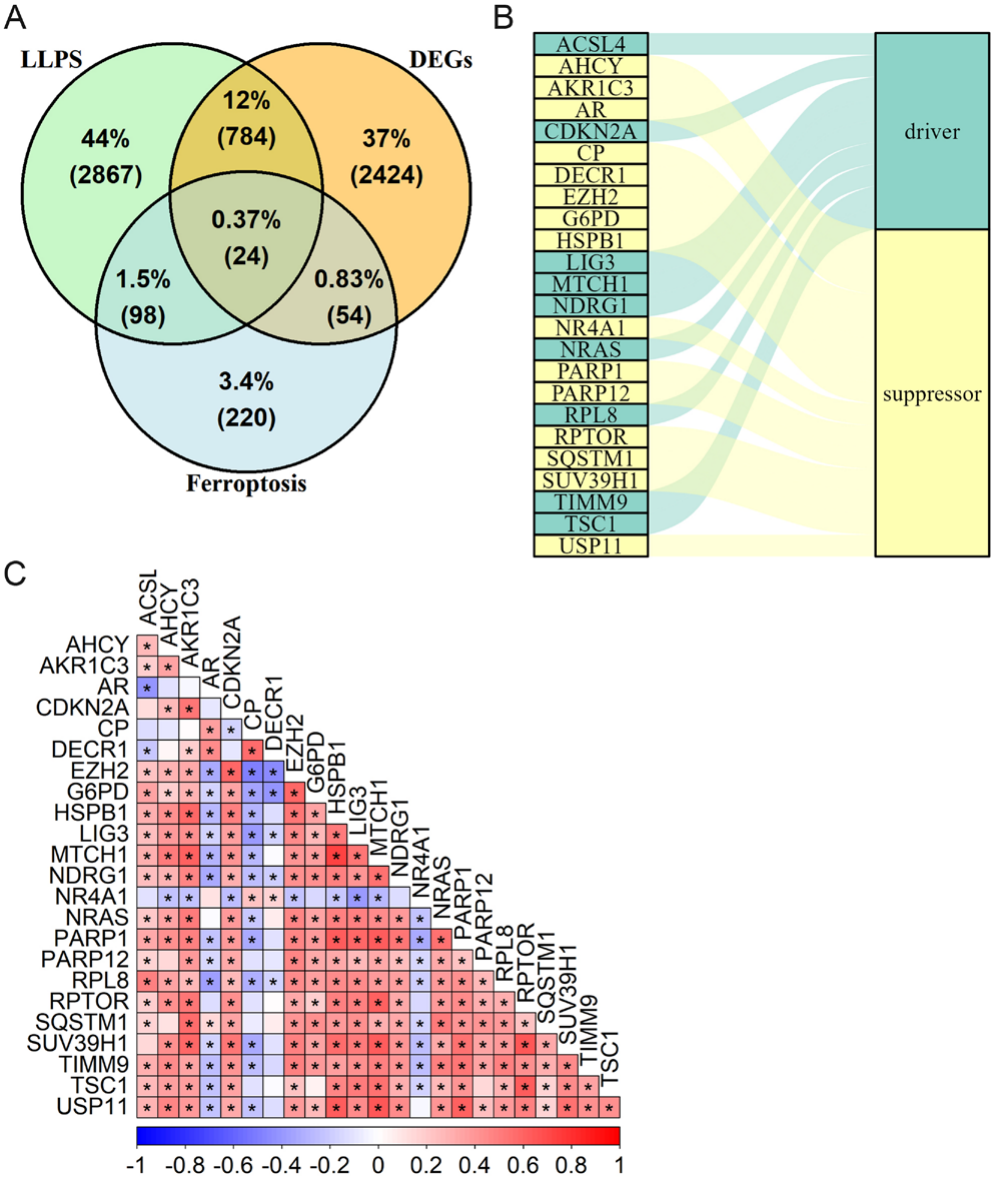
Identification of LLPS and ferroptosis-related DEGs in HCC. (a) Venn diagram showing the 24 common DEGs from HCC-DEGs, LLPS-related, and ferroptosis-related genes. (b) Sankey diagram categorizing 24 genes into 9 ferroptosis-driving and 15 ferroptosis-inhibitory regulatory factors. (c) Heatmap of Pearson correlation coefficients between the identified genes (^*^P < .05).

We extracted 3773 LLPS-related genes from the DrLLPS and PhaSepDB databases. Additionally, 396 ferroptosis-related genes were sourced from the FerrDb database. A total of 24 common DEGs were determined among the HCC-related DEGs, LLPS-related genes, and ferroptosis-related genes (Figure 2a). From the 24 genes we identified, 9 act as ferroptosis-driving regulatory factors and 15 function as ferroptosis-inhibitory regulatory factors (Figure 2b). Furthermore, we investigated the Pearson correlation between these genes (Figure 2c).

### PPI Network Construction and Hub Genes Identification

**Figure 3.**
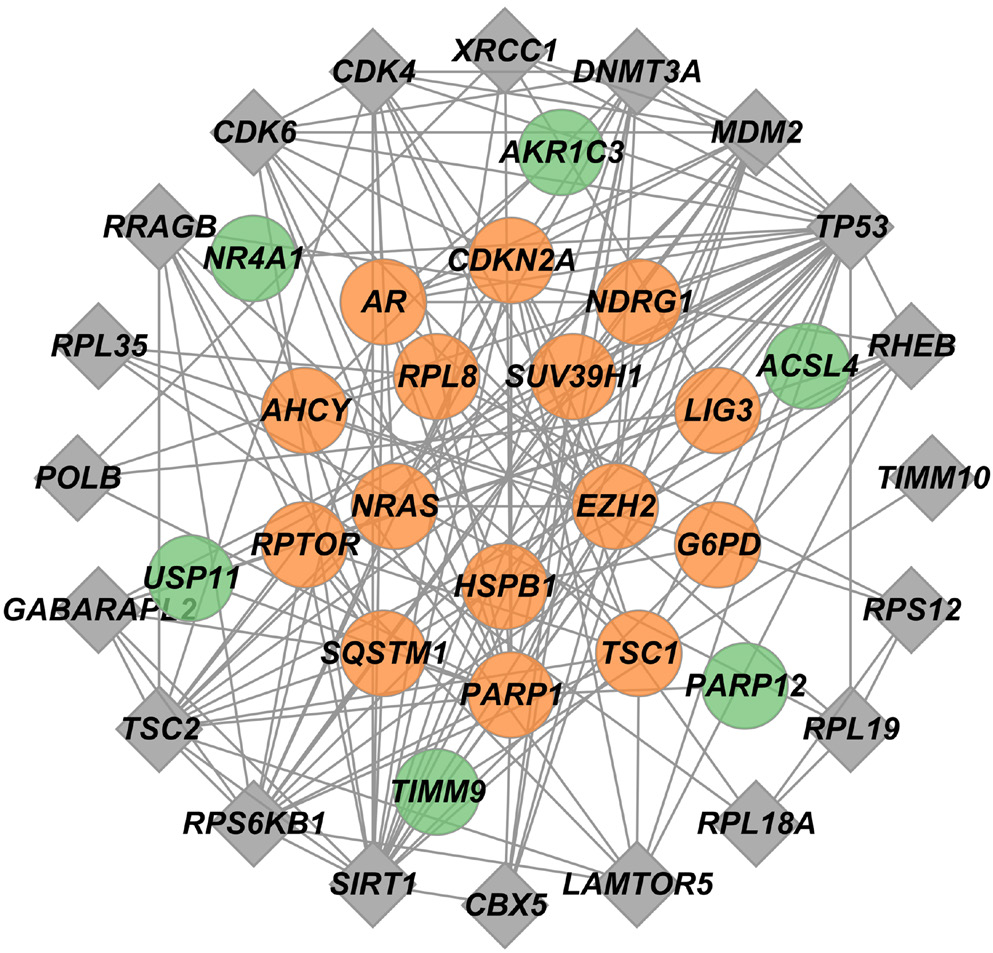
PPI network construction and hub gene identification. Within the network, genes from the 24 intersecting set are represented as circular nodes. Key genes, ranked by degree centrality, are highlighted in orange (top 15), while the remaining genes in this set are depicted in green. Genes predicted to have novel interactions are shown as square nodes.

The STRING database was employed to construct a PPI network, illustrating the salient interactions between proteins encoded by commonly identified DEGs. This PPI network, visualized using Cytoscape, comprised 41 nodes (Figure 3). Within this network, 21 out of the 24 pinpointed intersecting genes were included, while another 20 genes were predicted to potentially interact with different proteins therein. The central 15 hub genes identified were PARP1, CDKN2A, EZH2, AR, NRAS, TSC1, SQSTM1, RPTOR, SUV39H1, RPL8, NDRG1, LIG3, HSPB1, AHCY, and G6PD.

### Development of a Diagnostic Model

**Figure 4.**
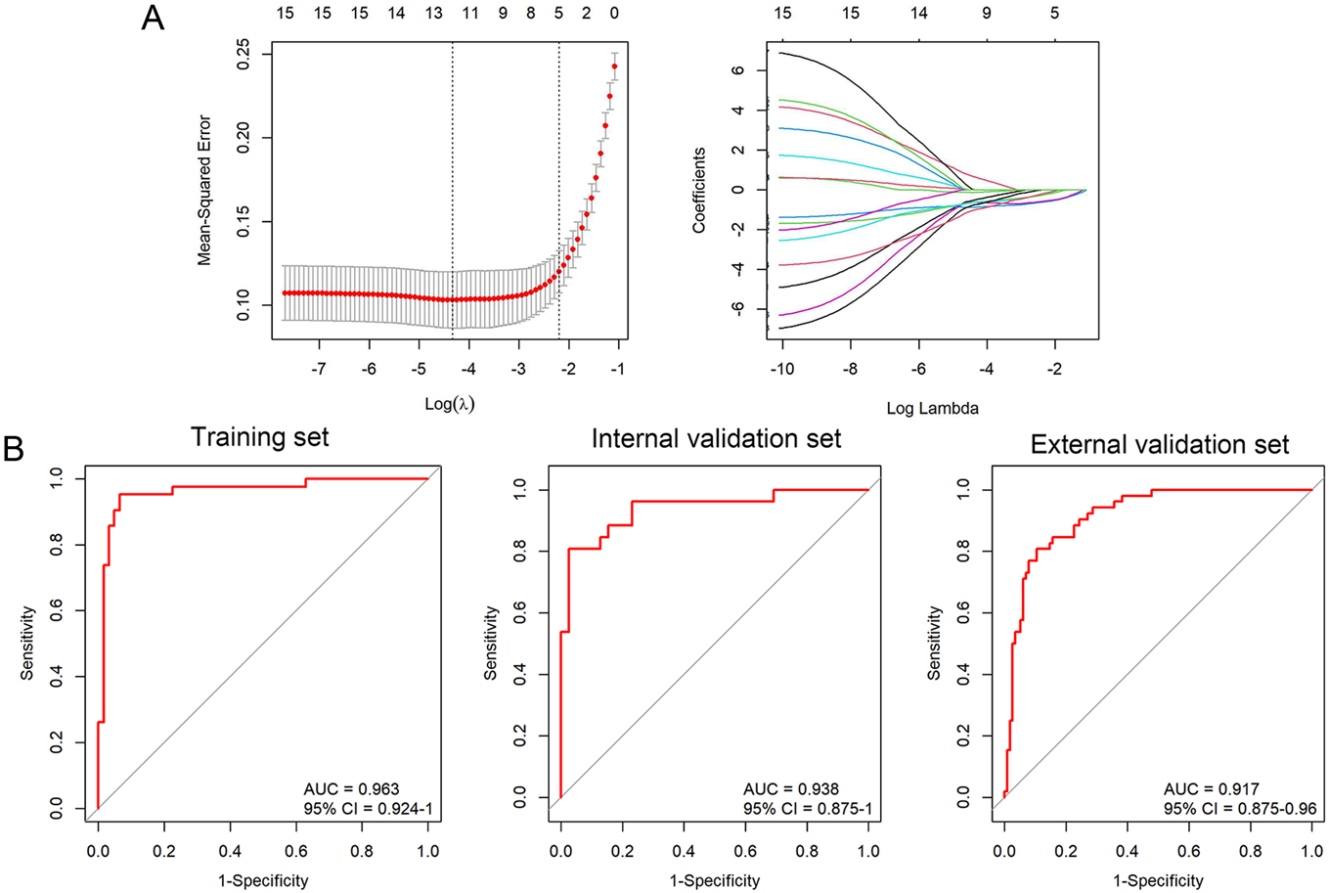
Development and evaluation of the prognostic model. (a) LASSO coefficient profiles of the 15 hub genes. (b) ROC curves for the training set (AUC = 0.963), internal validation set (AUC = 0.938) and external validation set (GSE76427) (AUC = 0.917).

According to the 15 hub genes derived from the PPI network, we applied the LASSO regression analysis for optimal feature selection (Figure 4a). This led to the formulation of a diagnostic model consisting of 5 hub genes: EZH2, HSPB1, NRAS, RPL8, and SUV39H1. We developed a diagnostic prediction model based on the following formula: prediction probability = 16.56 − 0.07*EZH2 − 0.81*HSPB1 − 0.05*NRAS − 0.39*RPL8 − 0.36*SUV39H1. To prevent overfitting, we allocated 60% of the dataset samples to the training set and evaluated the classification efficacy of the model on the subsequent 40% as an internal validation set. The diagnostic model exhibited areas under the ROC curve of 0.963 for the training set, 0.938 for the internal validation set, and 0.917 for the external validation set (GSE76427) (Figure 4b). Herein, the samples were divided into HCC and control groups with the optimal threshold of 0.468 in the training set, and the accuracy, recall sensitivity, precision, and specificity values were 0.9423, 0.9524, 0.9091, and 0.9355, respectively; in the internal validation set, the samples were divided into HCC and control groups with the optimal threshold of 0.512, and the accuracy, recall sensitivity, precision, and specificity values were 0.9077, 0.8077, 0.9545, and 0.9744, respectively; the samples were divided into HCC and control groups with the optimal threshold of 0.094 in the external validation set, and the accuracy, recall sensitivity, precision, and specificity values were 0.8683, 0.8077, 0.7778, and 0.8957, respectively.

### Independent Prognostic Value of 5 Signature Markers

**Figure 5.**
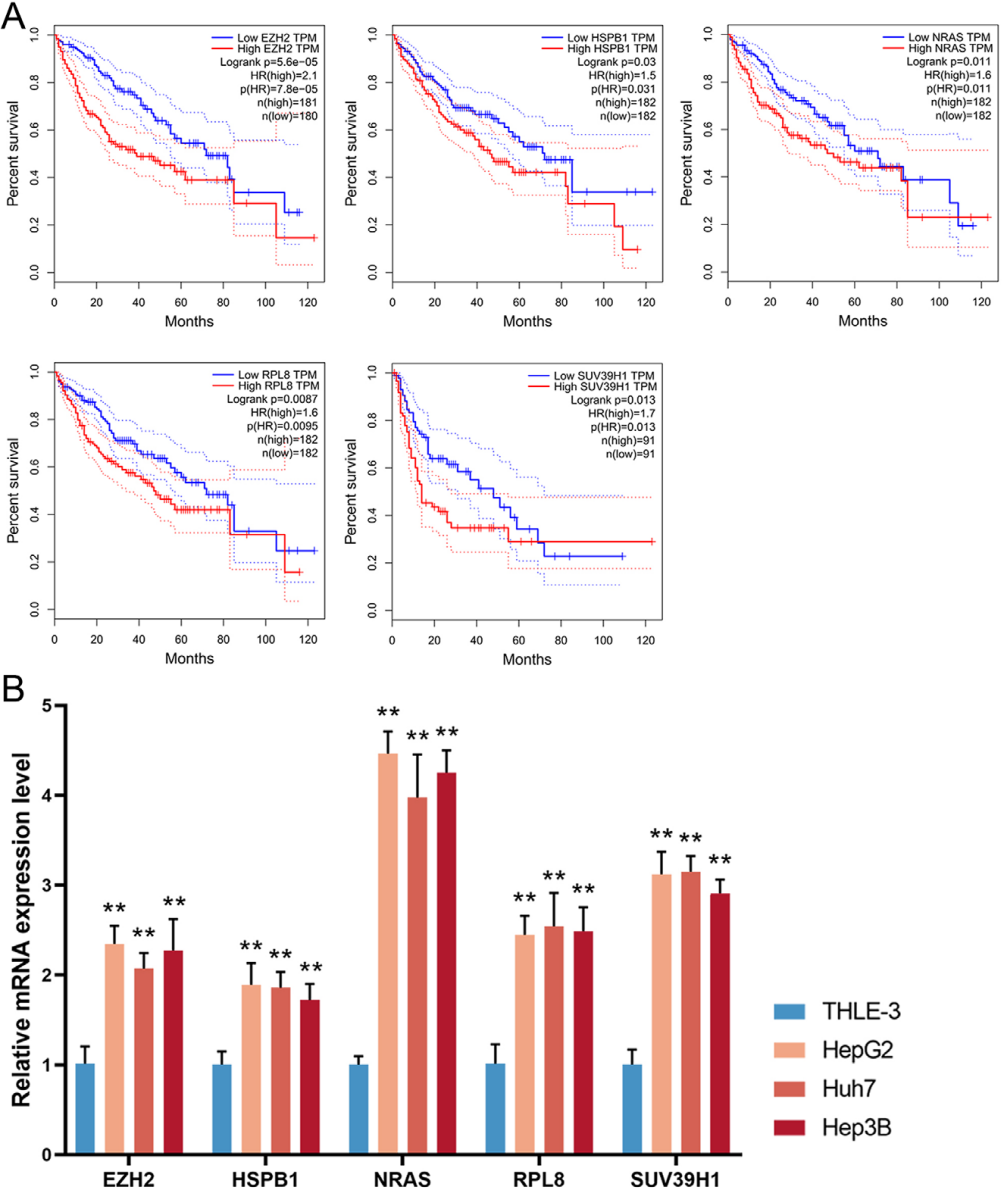
Prognostic significance and expression analysis of the 5 marker genes. (a) Kaplan-Meier survival curves comparing survival rates of patients based on gene expression levels. (b) The relative mRNA expression of EZH2, HSPB1, NRAS, RPL8, and SUV39H1 in HCC cell lines (HepG2, Huh7, and Hep3B) versus normal THLE-3 cells assessed via RT-qPCR (**P  <  .01).

From the KM survival analysis related to the 5 marker genes, it was observed that patients displaying lower expression levels of these genes consistently manifested higher survival rates in comparison to those with higher expression (*P* < .05, Figure 5a). This finding underscores the potential utility of these 5 markers (EZH2, HSPB1, NRAS, RPL8, and SUV39H1) as valuable prognostic tools for predicting disease outcomes. The expression levels of the 5 prognostic genes were determined using RT‒qPCR in normal THLE-3 cells and HCC cell lines (HepG2, Huh7, and Hep3B). As depicted in Figure 5b, there was a significant overexpression of EZH2, HSPB1, NRAS, RPL8, and SUV39H1 in HCC cells (*P* <  .01).

### NRAS Promotes Carcinogenesis of Hepatocellular Carcinoma Cells and Inhibits Ferroptosis

**Figure 6.**
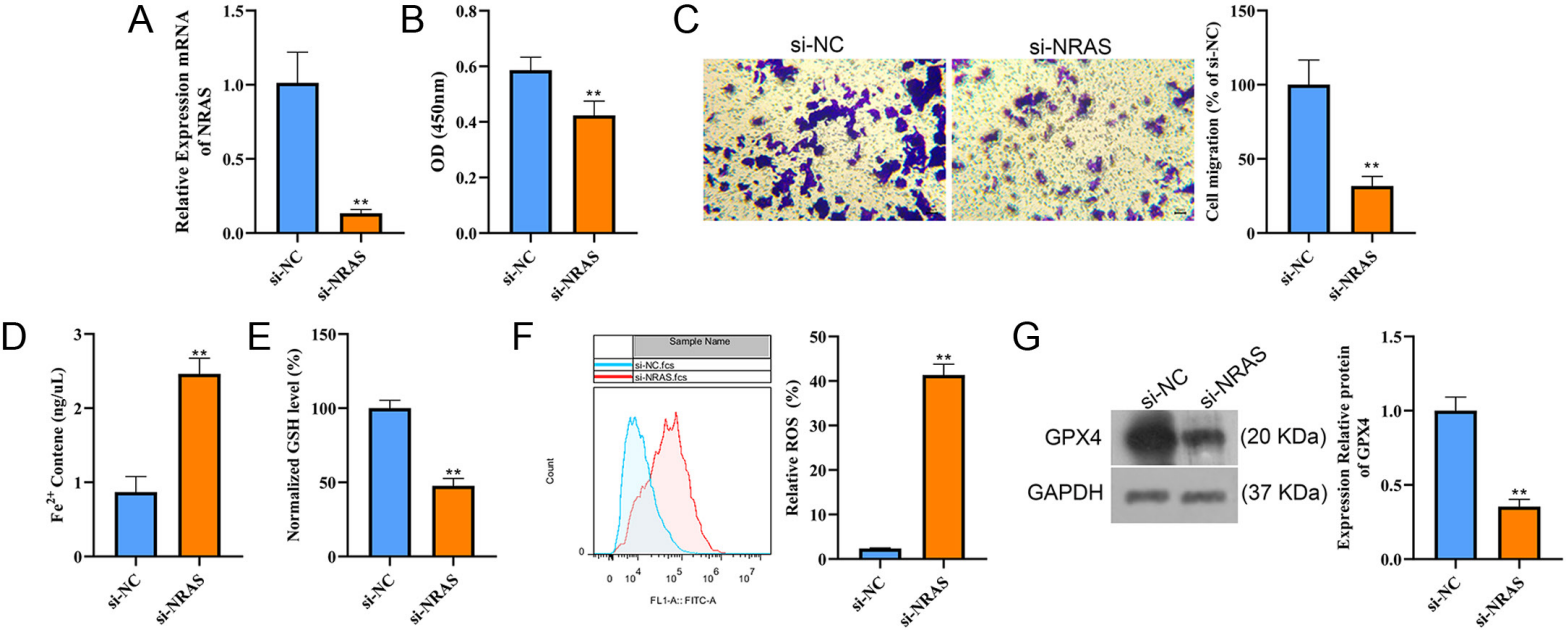
NRAS promotes carcinogenesis of HCC cells and inhibits ferroptosis. (a) Transfection efficiency was detected by RT-qPCR. (b) CCK8 assay detected HepG2 cell proliferation. (c) Transwell assay detected HepG2 cells migration. Fe2+ content (d) and GSH content (e) were efficiency was detected by RT-qPCR. (b) CCK8 assay detected HepG2 cell proliferation. (c) Transwell assay detected HepG2 cells migration. Fe2+ content (d) and GSH content (e) were detected. (f) ROS level was analyzed using flow cytometry. (g) Protein level of GPX4 was detected using western blot assay. **P  < .01.

To analyze the effect of the 5 genes in HCC, in vitro experiments were conducted. Compared to other genes, NRAS has the highest expression level in HepG2 cells (Figure 5b). Thus, NRAS and HepG2 were chosen for further study. To observe the effect of NRAS, the lentiviral vector of NRAS (si-NRAS) was constructed and transfected into HepG2 cells, and the transfection efficiency was detected by RT-qPCR (Figure 6a). Cell Counting Kit 8 assay and Transwell assay indicated that NRAS downregulation significantly inhibited HepG2 cell proliferation and migration (*P* < .01, Figure 6b and c). In addition, Fe^2+^ content and ROS levels were both significantly increased in the si-NRAS group when compared to those in the si-NC group (*P* < .01, Figure 6d and f), while opposite results were observed in GSH content and protein level of GPX4 (Figure 6e and g). These results demonstrated that NRAS promotes carcinogenesis of HCC cells and inhibits ferroptosis.

## Discussion

Liquid–liquid phase separation is pivotal in regulating the hallmarks of cancer, and previous studies have highlighted its significant role in the onset and progression of cancers, including HCC.^29,30^ However, the specific functions of LLPS in cancer remain elusive. Studies have shown that LLPS malfunction is instrumental in the anomalous accumulation of proteins such as α-syn and tau, and its interplay with iron metabolic imbalances is pivotal in driving ferroptosis.^14^ Ferroptosis, an iron-dependent form of regulated cell death, has recently emerged as a promising therapeutic approach to target cancer cells, especially those resistant to conventional treatments.^31-33^ Given that 81.7% of ferroptosis-related genes were differentially expressed in HCC, targeting ferroptosis could be a therapeutic strategy for HCC.^20^ Consequently, pinpointing LLPS and ferroptosis-associated biomarkers could yield significant insights into assessing the prognostic outcomes for HCC patients. In this study, we concentrated on HCC, delving into LLPS and ferroptosis-related patterns to deepen our comprehension of their roles in HCC pathogenesis. We established a 5-LLPS and ferroptosis genes-based diagnostic model for the prediction of patients with HCC.

The 5-gene diagnostic model we constructed encompasses EZH2, HSPB1, NRAS, RPL8, and SUV39H1. EZH2 belongs to the polycomb group gene family, pivotal epigenetic regulators known for repressing transcription.^34^ Wei et al^35^ observed an elevated expression of EZH2 in human HCC and mouse hepatoma tissues compared to their non-tumor counterparts. In line with this, we found that EZH2 expression was elevated in HCC cells. Furthermore, HCC patients with heightened EZH2 expression exhibited reduced survival rates. HSPB1, also known as HSP27, is a member of the small HSP family and has been shown to promote HCC metastasis via the Akt signaling pathway, potentially serving as a predictor for HCC patient outcomes.^36^ Additionally, HSPB1 has been identified as a negative regulator of ferroptosis, acting by reducing iron-mediated lipid ROS generation.^37^ Our analysis revealed that HSPB1 is also an LLPS-related DEG in HCC, underscoring its pivotal role in regulating both LLPS and ferroptosis within HCC. Overexpression of RPL8 enhances cell growth, mobility, and glycolytic activity in HCC.^38^ SUV39H1, a methyltransferase, utilizes SAM to drive H3K9me3 modification, suppressing the oncogene S100A11, leading to enhanced HCC progression.^39^ In addition, an upregulation of SUV39H1 was evident in both HBV-infected humanized mouse livers and clinical tissues of HBV-related HCC.^40^ We additionally noted an elevated expression of SUV39H1 in HCC cells, correlating with poorer survival outcomes in HCC patients. Besides, an RNA-seq analysis suggested that NRAS, as a potential target of TRERNA1, mediates certain aspects of hepatocellular carcinogenesis.^41^ Analysis of immune infiltration indicates a positive correlation between NRAS and the presence of CD68^+^ tumor-associated macrophages in HCC samples, with NRAS being linked to unfavorable HCC outcomes.^42^ These results suggested that these 5 genes may influence tumor progression by regulating LLPS and ferroptosis in HCC. Moreover, to analyze the effect of these 5 genes in HCC,in vitro experiments were conducted. Compared to other genes, NRAS has the highest expression level in HepG2 cells. Thus, NRAS and HepG2 were chosen for further study. The results found that NRAS downregulation significantly inhibited HepG2 cells proliferation and migration. In addition, Fe^2+^ content and ROS levels were both significantly increased in the si-NRAS group when compared to those in the si-NC group, while opposite results were observed for the protein level of GPX4 and GSH. These results demonstrated that NRAS promotes carcinogenesis of HCC cells and inhibits ferroptosis, which were consistent with bioinformatics analysis. However, the functions of the other 4 genes still need to be verified.

In both the training set, internal validation set, and external validation set (GSE76427), the diagnostic model demonstrated AUCs of 0.963, 0.938, and 0.917, respectively, exhibiting high sensitivity and specificity. In addition, survival curves indicated that higher expression of these 5 genes was associated with a poorer prognosis compared to lower expression. Moreover, we confirmed the expression levels of these 5 genes in HCC cell lines and normal cells using RT-qPCR. We found that all these 5 genes (EZH2, HSPB1, NRAS, RPL8, and SUV39H1) had higher expression levels in HCC cells, which were consistent with the results of survival analysis. These findings underscore the significant biological functions of these genes in HCC, related to LLPS and ferroptosis. Moreover, all validation outcomes affirm the accuracy and credibility of our constructed diagnostic model, which may be useful to guide the diagnosis of HCC in clinical applications. However, this diagnostic model might not be a useful tool to assess all patients with HCC because of the different etiology, disease stage, or treatment history of HCC patients. Thus, further in-depth research is needed to incorporate different conditions of HCC patients to verify the predictive performance of the diagnostic model. Moreover, a recent study highlighted the emerging role of prognostic markers to better select the systemic treatment for HCC.^43^ Quan et al^44^ found that loss of EZH2 confers resistance to tyrosine kinase inhibitors in non-small cell lung cancer. Musiani et al^45^ illustrated that as HSPB1 increase impairs the effectiveness of EGFR inhibitors and is known to protect cells from chemotherapeutics, the induction of HSPB1 by targeted agents might strongly affect the success of combination treatments. Treatment outcomes for NRAS-mutated and NRAS-wild type patients were investigated in 2 retrospective trials for MEK inhibitor and/or immune checkpoint inhibitors showing modest improvement.^46,47^ Thus, these 5 genes identified in this study might help to select the systemic treatment for HCC patients.

Our study has several limitations. Firstly, being retrospective, its findings might not carry the same weight as those from prospective studies. Furthermore, the modest sample size of both the training and validation datasets could introduce potential deviations. In addition, HCC is a biologically heterogeneous cancer (e.g., differences in etiology, disease stage, or treatment history); thus, the samples included in this study might affect the results obtained. To solidify our conclusions, more in vivo and in vitro experiments focusing on LLPS and ferroptosis genes in HCC are warranted.

We developed a unique 5-gene diagnostic model for HCC based on LLPS and ferroptosis-related genes, distinguishing it from previous signatures. To our knowledge, this is the inaugural effort to combine LLPS with ferroptosis genes in forecasting HCC patient outcomes. This signature underscores the therapeutic potential of targeting both LLPS and ferroptosis in cancer interventions. Additionally, our work hints at a potential link between LLPS and ferroptosis, paving the way for clearer insights into their combined impact on HCC prognosis.

## Availability of Data and Materials:

The datasets used and/or analyzed during the current study are available from the corresponding author on reasonable request.

## Supplementary Materials

Supplementary Material

## Figures and Tables

**Supplementary Figure 2.**
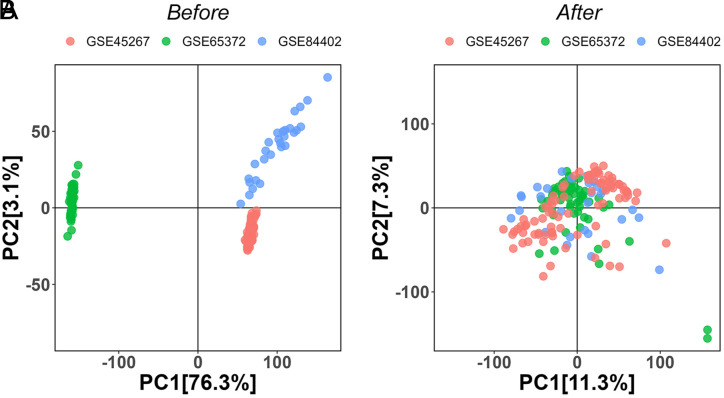
PCA dimensionality reduction of 3 HCC-GEO datasets (GSE45267, GSE65372, and GSE84402). (A) Before data integration, pronounced batch effects are shown. (B) After batch effect correction, a homogenized distribution is demonstrated.

**Table 1.** Details of Each Dataset from Gene Expression Omnibus Database

		GSE45267	GSE65372	GSE84402	GSE76427
Tissue	Tumor	48	39	14	115
	Normal	39	15	14	52
Age	>65 years	12	/	2	50
	≤65	75	/	24	65
Gender	Male	/	/	18	93
	Female	/	/	10	22

**Table 2.** Primers Used for Real-Time Quantitative Polymerase Chain Reaction

Genes	Primer (5’ to 3’)	Size (bp)
EZH2 (Human)	Forward: GCGGATAAAGACCCCACCAA	438 bp
	Reverse: GTATCCACATCCTCAGCGGG	
HSPB1 (Human)	Forward: GAGCTGACGGTCAAGACCAA	220 bp
	Reverse: TGGTGATCTCGTTGGACTGC	
NRAS (Human)	Forward: GTTGGGAAAAGCGCACTGAC	412 bp
	Reverse: CCTGTCTGGTCTTGGCTGAG	
RPL8 (Human)	Forward: GCCACCGTTATCTCCCACAA	135 bp
	Reverse: GGGTTTGTCAATTCGGCCAC	
SUV39H1 (Human)	Forward: CCCTGCCCTCGGTATCTCTA	276 bp
	Reverse: GCCTTCTGCACCAGGTAGTT	
GAPDH (Human)	Forward: GAAGGTCGGAGTCAACGGAT	133 bp
	Reverse: CTTCCCGTTCTCAGCCATGT	
