# Supplementary material for: Machine Learning Diagnostic Model for Hepatocellular Carcinoma Based on Liquid–Liquid Phase Separation and Ferroptosis-Related Genes
Source: Turk J Gastroenterol. 2024 Oct 7;36(2):89–99. doi: 10.5152/tjg.2024.24101 (PMC11843271; doi:10.5152/tjg.2024.24101)
Supplement: Supplementary Material [file supplementary_material.pdf]

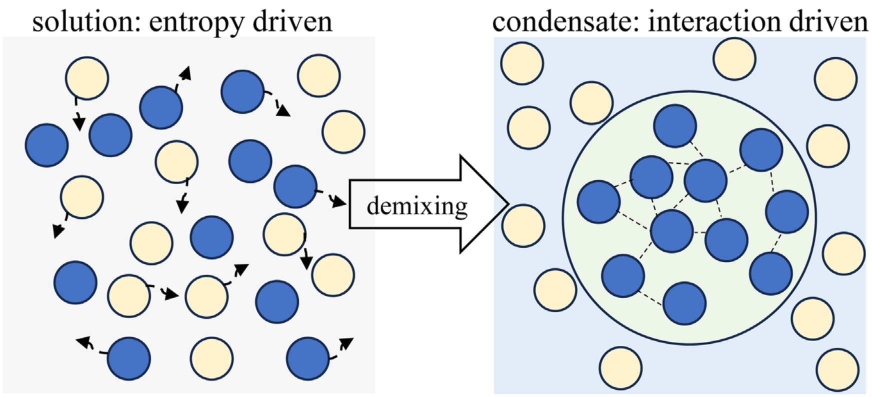

Supplementary Figure 1. liquid-liquid phase separation (LLPS). Whereas entropy typically drives molecules to become dispersed in solution, mutual interactions among a subset of molecules can shift the free-energy landscape to favor demixing and drive the formation of a separate condensed phase.

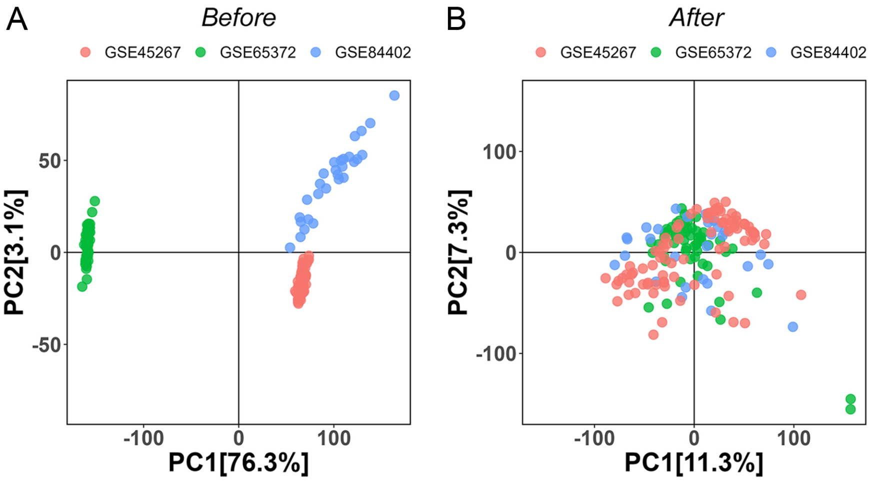

Supplementary Figure 2. PCA dimensionality reduction of 3 HCC-GEO datasets (GSE45267, GSE65372, and GSE84402). (A) Before data integration, pronounced batch effects are shown. (B) After batch effect correction, a homogenized distribution is demonstrated.
